# Diagnostic Value of Delayed PET/MR in Liver Metastasis in Comparison With PET/CT

**DOI:** 10.3389/fonc.2021.717687

**Published:** 2021-08-30

**Authors:** Nina Zhou, Xiangxi Meng, Yan Zhang, Boqi Yu, Jianmin Yuan, Jiangyuan Yu, Hua Zhu, Zhi Yang

**Affiliations:** ^1^Key Laboratory of Carcinogenesis and Translational Research (Ministry of Education/Beijing), NMPA Key Laboratory for Research and Evaluation of Radiopharmaceuticals (National Medical Products Administration), Department of Nuclear Medicine, Peking University Cancer Hospital & Institute, Beijing, China; ^2^Central Research Institute, United Imaging Healthcare Group, Shanghai, China

**Keywords:** liver metastasis, PET/CT, PET/MR, comparative study, delayed PET scan

## Abstract

**Objectives:**

The aim of this study was to evaluate the value of a delayed positron emission tomography/magnetic resonance (PET/MR) scan relative to a single positron emission tomography/computed tomography (PET/CT) scan for liver metastasis detection.

**Methods:**

In this study, 70 patients with solid malignancies and suspicious liver lesions undergoing 2-deoxy-2-[^18^F]fluoro-*D*-glucose [(^18^F)FDG] PET/CT and subsequent delayed liver PET/MR scans were analyzed. The histopathological analysis and/or imaging follow-up were performed as the standard of reference. Lesion maximum standardized uptake value (SUVmax), diameter, and tumor to nontumor ratio (T/N) were measured. Lesion detection sensitivity, specificity, positive predictive value (PPV), and negative predictive value (NPV) were calculated for both examinations.

**Results:**

(1) The standard of reference revealed 208 liver lesions in 70 patients (metastasis in 56 patients with 196 lesions; benign in 14 patients with 12 lesions). Compared with PET/CT, PET/MR had higher accuracy (98.6% *vs.* 78.6%), sensitivity (98.2% *vs.* 76.8%), and specificity (100.0% *vs.* 85.7%) (2). The therapeutic strategies of 29 patients (41.4%) needed reconsideration after the additional PET/MR, including new metastases detected (13/70), new affected lobes identified (14/70), and false-positive corrected (2/70) (3). PET/MR detected significantly more metastases than PET/CT did, especially with small lesions. The SUVmax of the same lesion correlated well between the two acquisitions, while the delayed PET showed a higher T/N ratio.

**Conclusions:**

In liver metastasis detection, the diagnostic value of the delayed PET/MR is validated to be superior to that of PET/CT, which may aid the clinical decision-making.

## Introduction

Clinical management of patients with malignant liver lesions requires advanced diagnostic and therapeutic methods. Thus, medical imaging has been profoundly integrated into the clinical decision-making and patient care processes in cases of primary and metastatic liver cancers (1, 2). Currently, positron emission tomography/computed tomography (PET/CT) has been widely used in solid neoplasm detection and staging (3), including liver malignancies (4), demonstrating higher accuracy in the tumor, node, metastasis (TNM) staging than the single modality of CT or PET (5, 6). However, the limitations of PET/CT have been exposed as clinical evidence accumulates. For one thing, the soft-tissue contrast of CT is not optimal for diagnosis and differentiation of soft-tissue lesions. For another, PET alone is not enough to draw conclusions on tumors with low metabolic activities, such as well-differentiated hepatocellular carcinoma, neuroendocrine tumors, and mucinous adenocarcinoma (7–9).

In the technical aspect, the image quality of traditional PET scanners also limits the detectability of lesions of small sizes and relatively low contrast, such as small lymph nodes, subcentimeter liver lesions, and even larger lesions of clear cell renal cell carcinoma (10–12). Without consulting other registered modalities, these lesions with underlying clinical significance are easily overlooked even by experienced radiologists, leading to false-negative diagnosis.

In 2011, combined PET and magnetic resonance imaging hybrid units (PET/MR) were approved in both the USA and the European Union (13). Since then, PET/MR scans have been recommended to patients, alone or as a delayed scan after the initial PET/CT examination. Up to now, the potential value and role of PET/MR in clinical practice is yet to be established (14, 15). MR imaging has a superior soft tissue contrast compared with CT, which enables detailed evaluation of soft tissues within the abdomen, pelvis, and central nervous system (16, 17). Moreover, MR imaging affords the opportunity to evaluate tissue function with dedicated sequences including diffusion-weighted imaging (DWI), MR spectroscopy, and perfusion-weighted imaging. These types of information acquired by MR, combined with the metabolic information from PET, reflect the physiological characteristics of the lesion more accurately, leading to higher diagnostic efficiency (7).

As a widely accepted medical imaging examination in cancer diagnosis, MR, often accompanied by the administration of contrast media, is able to delineate anatomical features of tissues, including margins, local infiltration, and the relationship of tumors to adjacent structures, and has shown a comparative advantage over CT (18–20). On the other hand, the use of MR is hampered by several factors, including lesion properties such as lesion size and lesion location, as well as technical limitations, such as the spatial resolution, motion artifacts, and susceptibility artifacts. Previous studies have shown that the sensitivity and specificity of MR imaging in lesion detection is correlated to the size of lesions (20–22). Owing to the low image contrast of small lesions, PET also shows an inferior detection ability in such a situation. Taking advantage of the combination of the superior soft-tissue contrast of MR and the molecular imaging of PET, PET/MR can be used to detect small liver lesions, and might help to differentiate their benign or malignant nature. Therefore, the purpose of this work is to evaluate the value of a delayed PET/MR without contrast enhancement compared with PET/CT for liver metastasis detection.

## Materials and Methods

### Patient Enrollment

This study was performed under a single-center prospective imaging protocol and approved by the Medical Ethics Committee of Peking University Cancer Hospital (ethical approval no. 2018KT110-GZ01). All patients provided written informed consent before the study participation. Patient recruitment was performed between Oct 2019 and Aug 2020. Patients referred for PET scans to confirm the suspicious liver lesions were enrolled for an additional delayed PET/MR after the initial 2-deoxy-2-[^18^F]fluoro-*D*-glucose ([^18^F]FDG) PET/CT scan. In this study, the inclusion criteria for the study participation included any of the following conditions: (a) PET/CT detection of liver metastasis in two lobes, the number being no more than three; (b) PET/CT detection of less than five liver metastases, all in one lobe; (c) CT detection of low-density, non-[^18^F]FDG avid liver nodules; and (d) suspicious metastases detected by previous medical exams but missed in the PET/CT scan. However, patients with any of the following conditions were excluded: (a) pregnancy; (b) age <18 years old; (c) inadequate PET/CT images, due to artifacts, system malfunction, or poor patient cooperation; (d) contraindication to MR imaging; and (e) inability to tolerate the PET/MR imaging. After enrollment, patient with any of the following conditions were excluded from data analysis: (a) not completing the PET/MR scan, (b) inadequate PET/MR images, and (c) insufficient follow-up to confirm the reference standard.

### [^18^F]FDG PET/CT Imaging

Imaging was performed using a PET/CT scanner (Biograph64, SIEMENS, Erlangen, Germany) operated in 3D Flow Motion (bed entry speed 1 mm/s) over an axial field of view from the apex of the skull to the mid-thigh. Low-dose CT scans were acquired in the CARE Dose4D mode (120 kV, image slice thickness, 3.0 mm). The patients were instructed to fast for at least 6 h before [^18^F]FDG injection. In all cases, the serum glucose concentration met the institutional requirement (≤140 mg/dl). The injected activity was 3.7 MBq/kg, and the time from injection to scan was 60 min.

A three-dimensional ordered-subset expectation maximum (3D OSEM) algorithm (2 iterations and 21 subsets) with TrueX+true positive fraction (TPF) method was used to reconstruct PET images. The reconstruction voxel spacing was 4.1 mm × 4.1 mm, the slice thickness was 3 mm, and the matrix was 200 × 200. A 3-mm full width at half maximum (FWHM) Gaussian smoothing filter was applied.

### PET/MR Protocols

[^18^F]FDG PET/MR of the abdomen was performed on an integrated 3.0-T time-of-flight PET/MR scanner (uPMR790, United Imaging Healthcare, Shanghai, China). Each patient underwent the same protocol. The scan started 142.9 ± 23.9 min (range: 120–180 min) after [^18^F]FDG administration. The body array coil was placed around the individual and covered the entire liver. Respiratory gating was used in MR acquisition whenever possible. The MR sequences were preformed simultaneously during PET acquisition, including T2-weighted image with fat saturation (T2WI), T1-weighted image (T1WI), and DWI. The mean scan time for PET/MR was 20 ± 6 min. The detailed MR parameters are shown in **Table 1**.

**Table 1:** Acquisition parameters for the applied MR sequences.

Sequence	TR (ms)	TE (ms)	Matrix	FOV (mm)	Thickness (mm)	Gap (mm)	Fat Sat
**WFI with trigger**	5.06	2.24	256 × 329	350 × 500	4	0	NA
**T2WI FSE with fat saturation and trigger**	4,000^a^	88.74	320 × 177	380 × 300	6	1.2	Yes
**DWI (*b* = 50, 800 s/mm^2^)**	4,000	70	128 × 101	380 × 300	6	1.2	Yes
**T1WI with radial acquisition**	3.56	1.59	320 × 320	400 × 400	4	0	Yes
**Dual echo T1WI with breathhold**	4.22	2.58	320 × 168	400 × 300	6	0	NA

a T2WI with fat saturation sequence uses respiratory gating, TR differs in patients due to different respiratory rate. WFI, water fat imaging; FSE, fast spin-echo.

PET reconstruction was conducted with a 3D OSEM algorithm (2 iterations and 20 subsets), in a 256 × 256 matrix and smoothed by a Gaussian filter with 3 mm FWHM. The voxel spacing was 2.3 mm × 2.3 mm, and the slice thickness was 2.8 mm. A four-compartment-model attenuation map (μ-map) was automatically generated based on a water-fat-imaging sequence with breath gating and used for attenuation correction.

### Image Analysis

Lesion identification and patient diagnosis were performed based on the PET/CT and PET/MR images, according to the consensus of two accredited readers with experiences in hybrid imaging and MR of 4 and 6 years. In PET/CT, lesions were rated as metastases when PET showed positive uptake foci with or without hypodensity nodule on CT. On PET/MR, lesions were rated as metastases when at least two of the three following criteria were met: (a) hyperintense on T2WI, (b) diffusion restriction on DWI, and (c) PET positive. The PET/CT and PET/MR images of the same patient were evaluated separately, but the patient history was not blinded.

All lesions detected on PET/CT and PET/MR were documented for patients with less than 10 lesions. The smaller 10 visible lesions were recorded for those with more than 10 lesions. The properties of the lesions were then documented. For each detected lesion, the maximum single-voxel standardized uptake value (SUVmax) was calculated based on a spherical volume of interest (VOI) in the corresponding PET modality. The size of the lesion is represented by the short-axis diameter on T2WI MR images for all lesions measurable. The tumor-to-nontumor (T/N) ratio was determined based on a measurement of the liver background SUVmax. The lesions detected on PET/CT and PET/MR were paired according to the relative liver location, whenever possible.

### Reference Standard and Follow-Up

A combination of biopsy, surgical pathological analysis, correlation with prior imaging findings, and clinical and imaging follow-up was used as the reference standard for the liver lesions. Histopathologic analysis of biopsy samples or surgical pathologic analysis was used as the gold standard in determining the lesion identity, but they were not practical for all cases because of technical considerations, ethical considerations, or both. In these subjects, follow-up images (e.g., contrast enhanced MR, contrast enhanced CT, and/or follow-up PET/CT, showing continued tumor growth by the Response Evaluation Criteria in Solid Tumors), a comprehensive analysis of follow-up examinations and clinical manifestations (e.g., identification of new metabolically active lesions, lesions response to certain treatments), or both, were used to assess the lesion identity. The follow-up was conducted at least 60 days after the initial PET/CT and PET/MR studies.

### Statistics

Statistical analysis was performed using Statistical Product and Service Solutions (SPSS), version 26.0 (SPSS Inc.), and Origin 2019 (OriginLab Corporation). A patient-based and a lesion-based data analyses were performed. Sensitivity, specificity, accuracy, positive predictive value (PPV), and negative predictive value (NPV) were calculated. Results from the PET/CT and PET/MR were compared using two-tailed, unpaired Student’s *t*-tests and the McNemar square test. Linear regression has been conducted for paired measurement data, where Pearson’s *r* was used to evaluate the correlation. A *p-*value of 0.05 or less was considered statistically significant. The results were presented as the mean ± SD. Subsequently, these data were summarized using descriptive statistics.

## Results

### Patient Characteristics

**Figure 1:**
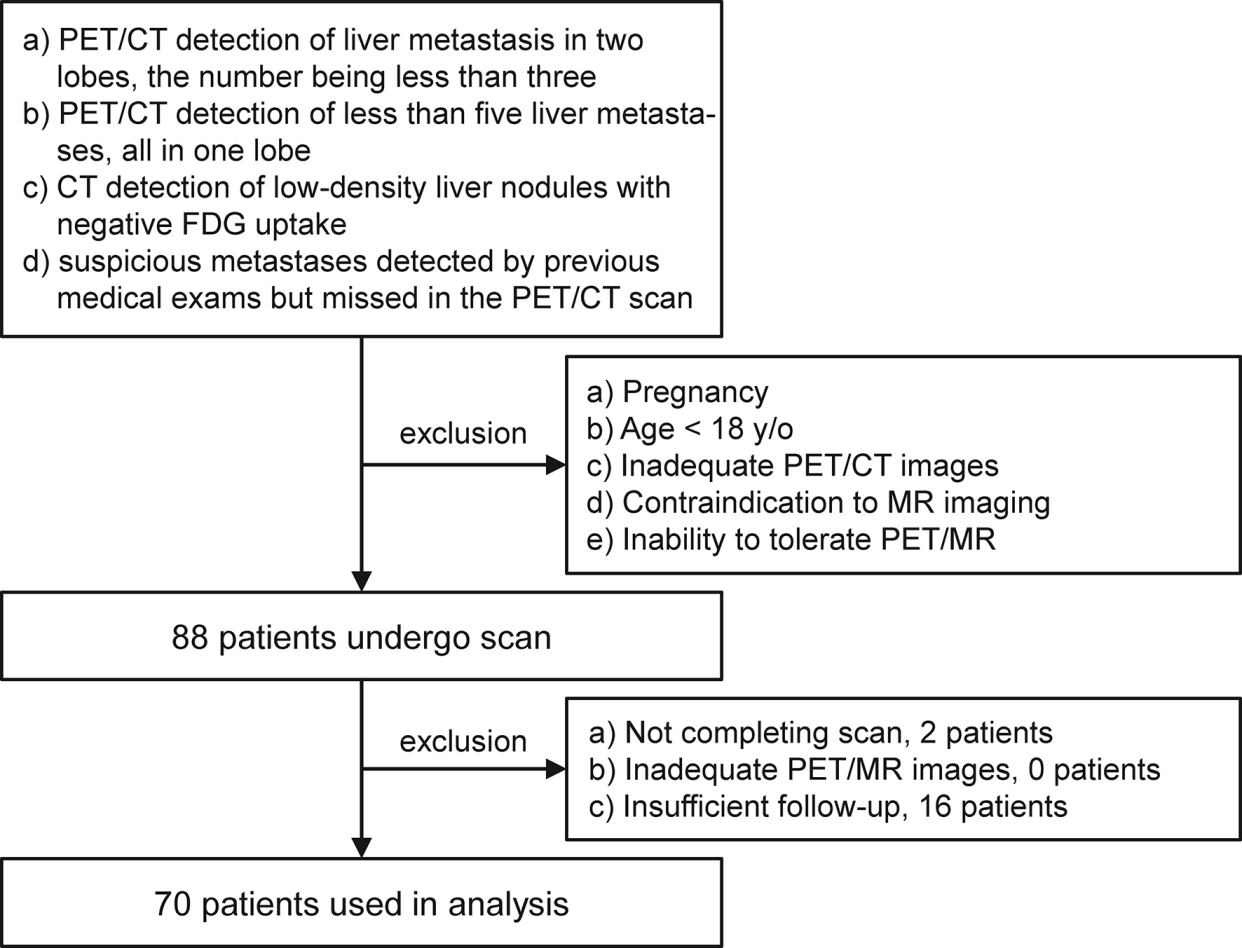
The enrollment and exclusion criteria.

Out of the 88 patients initially enrolled, only 70 were used in the analysis. Two patients were excluded for an incomplete delayed PET/MR scan, and another 16 were excluded for insufficient follow-up. The PET/MR image quality for all other patients met the quality control criteria (**Figure 1**). The median age of these 70 patients was 62 years old (26–84 years old). According to the reference standard (surgical pathology 7/70, contrast enhanced MR 19/70, contrast enhanced CT 22/70, PET/CT 2/70, comprehensive follow-up 20/70), liver metastases were present in 56 patients and benign lesions were present in 12 patients. The other two patients were confirmed to have no liver lesion. A total of 196 liver metastasis lesions and 12 benign lesions were detected. The patient characteristics are summarized in **Table 2**.

**Table 2:** Patient characteristics.

Patients characteristic	Number
Age (years)	26–84 (median 62)
Male/Female	38/32
Primary tumor	
Colon cancer	16
Lung cancer	10
Pancreatic cancer	9
Gastric cancer	9
Cholangiocarcinoma	4
Rectal cancer	12
Hepatocellular carcinoma	2
Melanoma	3
Breast cancer	2
Esophageal cancer	2
Germ cell tumor of testis	1
Newly diagnosed	37
Follow-up after surgery	33
In therapy	9
No therapy within 6 months	24
Follow-up interval (months)	2–10 (mean, 5.4)
Liver metastasis	56
Number less than 5	42
Number more than 5	14
In 1 lobe	40
In 2 lobes	16
Benign lesions	12
Cyst	3
Hemangioma	4
Other	5
No lesion	2

### The Diagnostic Power of PET/CT and PET/MR

Based on the reference standard, 56 patients had liver metastases and the other 14 patients had no lesions (*n* = 2) or benign lesions (*n* = 12).

PET/CT detected all metastases in 17/56 patients; it missed at least one metastasis in the other 39 metastatic patients, in which 13 cases resulted in false-negative diagnosis. Among the 14 cases without metastasis according to the reference method, two patients had false-positive [^18^F]FDG uptake and no identifiable lesion in CT; 12 had low-density lesions in CT but no elevated [^18^F]FDG uptake.

PET/MR detected metastasis in 55/56 patients, ruled out lesions in two patients showing false-positive PET uptake in PET/CT, and diagnosed benign lesions in 12 patients. PET/MR detected all lesions in 52/56 patients with liver metastases; in the remaining four patients, PET/MR missed at least one lesion, leading to one case of false-negative diagnosis.

**Figure 2:**
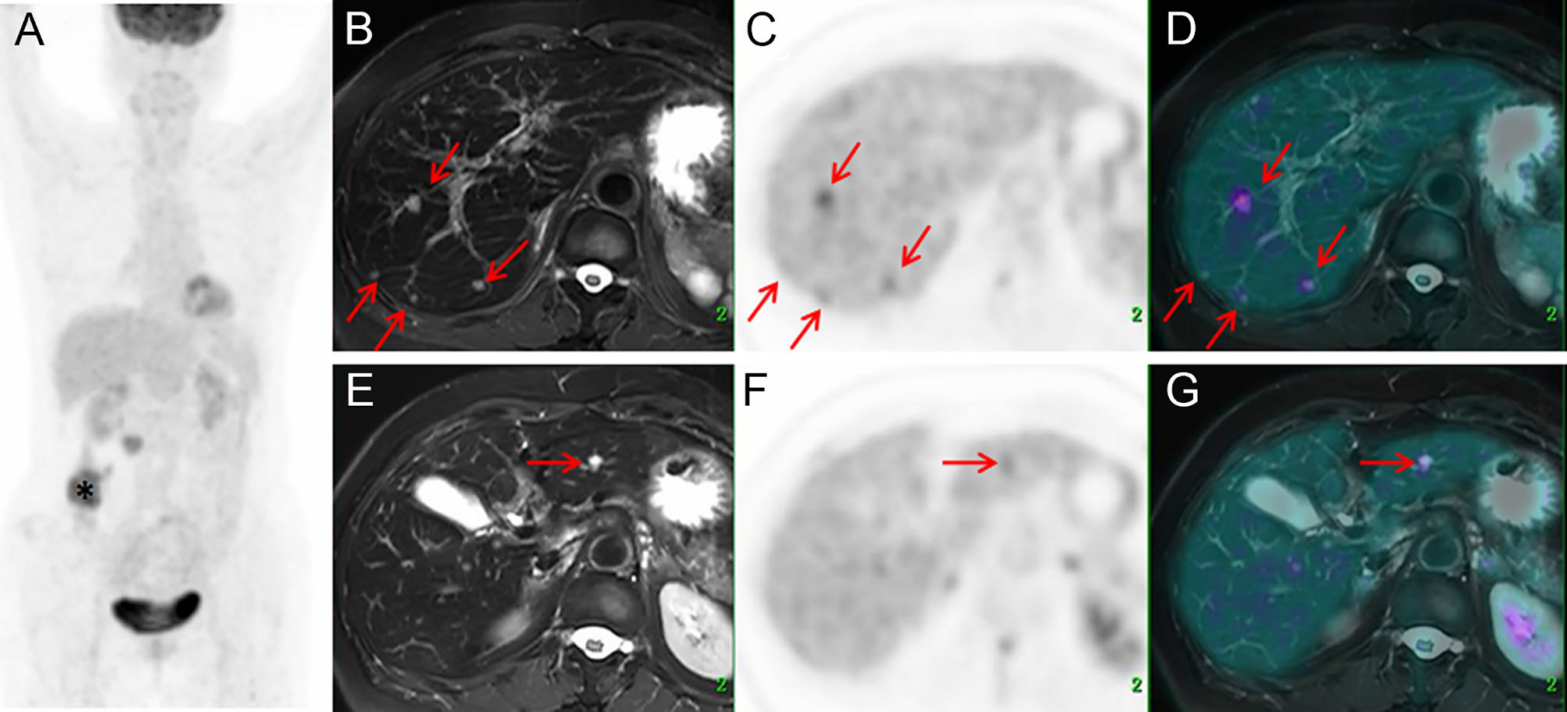
Images of a 64-year-old female with colonic mucinous adenocarcinoma. The MIP **(A)** of PET/CT showed a primary lesion at the ascending colon (*) with no [^18^F]FDG foci on the liver. Delayed T2WI, PET, and PET/MR-merged images showed multiple metastases (red arrow) with hyperintensity and hypermetabolism on the right lobe **(B–D)** and left lobe **(E–G)**.

Of the 56 patients with liver metastases, PET/MR showed an equal number of lesions with PET/CT in 18 cases, including one case with which both PET/CT and PET/MR made false-negative diagnosis. In all other 38/56 cases, PET/MR detected more metastases than PET/CT, including 13 cases with no metastasis detected on PET/CT (**Figure 2**). Furthermore, out of those 30 patients diagnosed with PET/CT as being affected by metastasis in only one liver lobe, 14 were confirmed by PET/MR as having metastases in more than one lobe.

A total of 196 metastatic lesions and 12 benign lesions (three cysts, four hemangiomas, and five other types) have been confirmed according to the reference standard. Of the metastatic lesions, 83/196 have been identified by PET/CT and 192/196 have been identified by PET/MR. PET/CT and PET/MR identified 2 and 0 false-positive lesions which were later confirmed as benign lesions or normal findings, respectively.

The diagnostic effectiveness based on patients as well as lesions is summarized (**Table 3**), including the sensitivity, specificity, accuracy, PPV, and NPV.

**Table 3:** Diagnostic effectiveness of PET/CT and PET/MR.

	Sensitivity (%)	Specificity (%)	Accuracy (%)	PPV (%)	NPV (%)
**In 70 patients**					
PET/CT	76.8	85.7	78.6	95.6	48.0
PET/MR	98.2	100	98.6	100	93.3
**In 208 lesions**					
PET/CT	35.2	85.7	38.6	97.2	8.6
PET/MR	98.0	100	98.1	100	75

### Detailed Analysis Based on Lesion Characteristics

**Figure 3:**
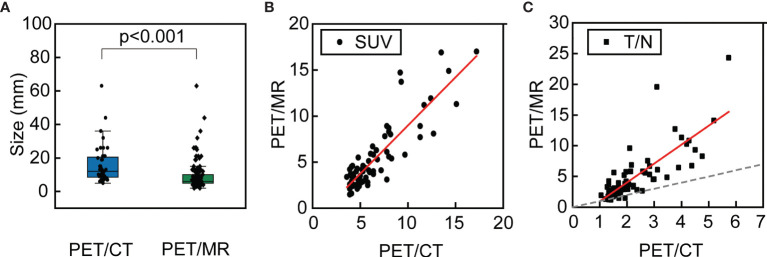
The box plot of the sizes of all metastases detected by PET/CT and PET/MR **(A)**; the relationship of the SUVmax of the same lesion detected by PET/CT and PET/MR **(B)** and the relationship of the T/N of the same lesion detected by PET/CT and PET/MR, where the dashed line indicates equal T/N **(C)**.

According to the McNemar test, for each patient, PET/MR detected significantly more liver metastases than did PET/CT (*p* < 0.001). A box plot was used to compare the lesion size distribution of all metastases detected by PET/CT and PET/MR (**Figure 3A**). Evidently, the mean lesion size detected by PET/MR was significantly smaller than that of PET/CT (*p* < 0.001).

For those lesions which were simultaneously identified on the PET modalities of PET/CT and PET/MR (*n* = 68), further analysis has been conducted. The corresponding liver background (3.0 ± 0.4 *vs.* 1.3 ± 0.3, *p* < 0.001) and lesion uptake (6.6 ± 3.2 *vs.* 3.6 ± 3.4, *p* < 0.001) were higher in PET/CT than in delay PET/MR. However, the T/N was higher in PET/MR than PET/CT (5.0 ± 4.2 *vs.* 2.3 ± 1.1, *p* < 0.001). The relationship of SUVmax and T/N has been plotted as scatter plots of PET/MR against PET/CT. As for SUV, it followed a linear relationship (Pearson’s *r* = 0.87) where the slope is 1.05 ± 0.07 (**Figure 3B**). As for T/N, it also roughly followed a linear relationship (Pearson’s *r* = 0.79), and it is evident that the T/N in PET/MR was mostly higher than those of PET/CT, with the slope of 3.08 ± 0.29 (**Figure 3C**).

**Figure 4:**
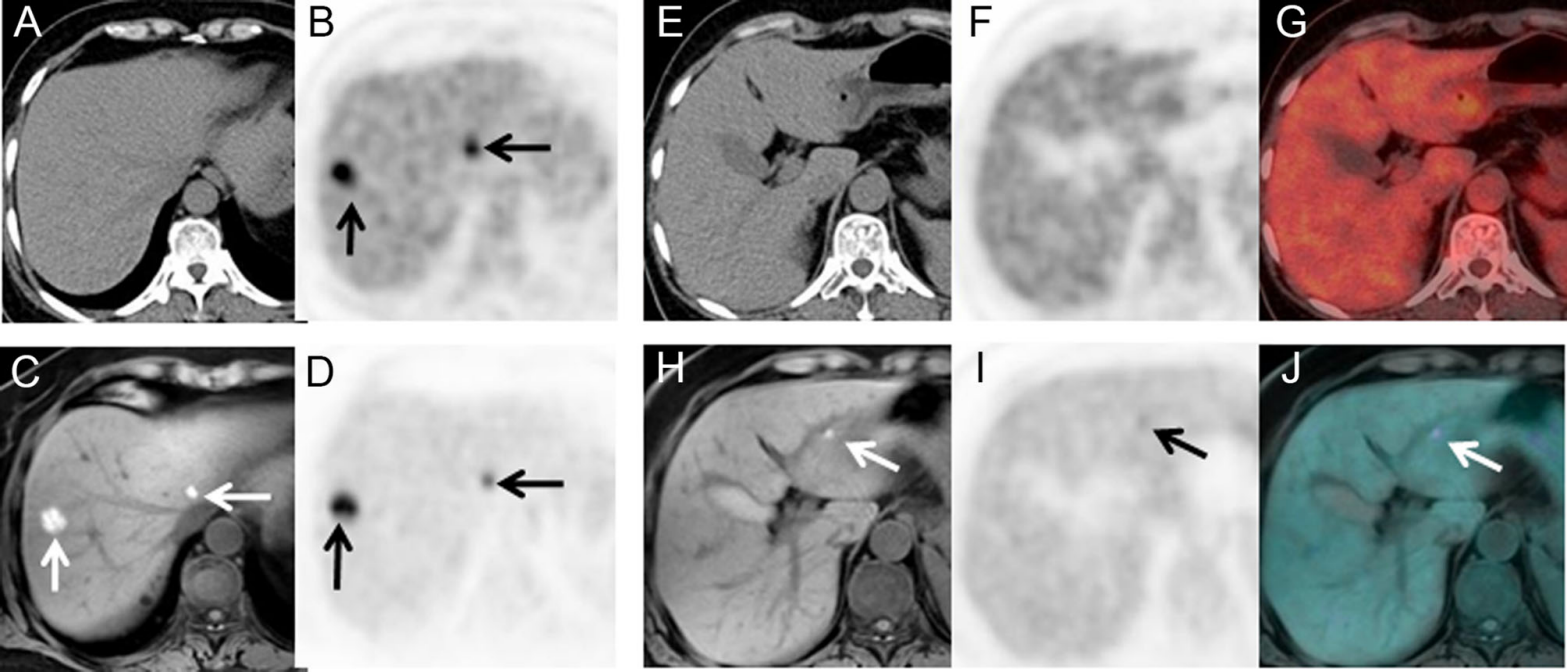
A 66-year-old female with surgical resection of malignant melanoma on head skin. She received PET/CT scans as regular follow-ups. Only two lesions were seen on PET/CT **(A, B)**. No lesion was seen on the left lateral lobe **(E–G)**. PET/MR clearly showed two lesions with hyperintensity on T1WI and hypermetabolism **(C, D)**, and an additional 4-mm small lesion was identified on T1WI and PET **(H–J)**, which only showed mild FDG uptake (T/N = 1.3).

Based on T2WI, DWI, and PET, 178/192 lesions were detected, although 44.3% (79/178) lesions among them had mild to moderate uptake (T/N ≤ 1.5) on the delayed PET images. Combined with T2WI+DWI or T1WI+DWI, these lesions were correctly diagnosed as metastasis (**Figure 4**). A total of 14 lesions in six patients were seen on T2WI+DWI without notable [^18^F]FDG uptake, and all of them had other definite lesions in the liver (**Table 4**).

**Table 4:** The part of PET/MR showing the lesion property in liver metastases detection.

The part of PET/MR showing lesion property	*N* (%) (*n* = 196)
PET/MR positive	
T2WI+DWI/T1WI+DWI	14 (7.1%)
T2WI+DWI+PET/T1WI+DWI+PET	178 (90.8%)
PET/MR negative	
T2WI	4 (2.0%)

### Further Detailed Evaluation Based on Clinical Indication

**Figure 5:**
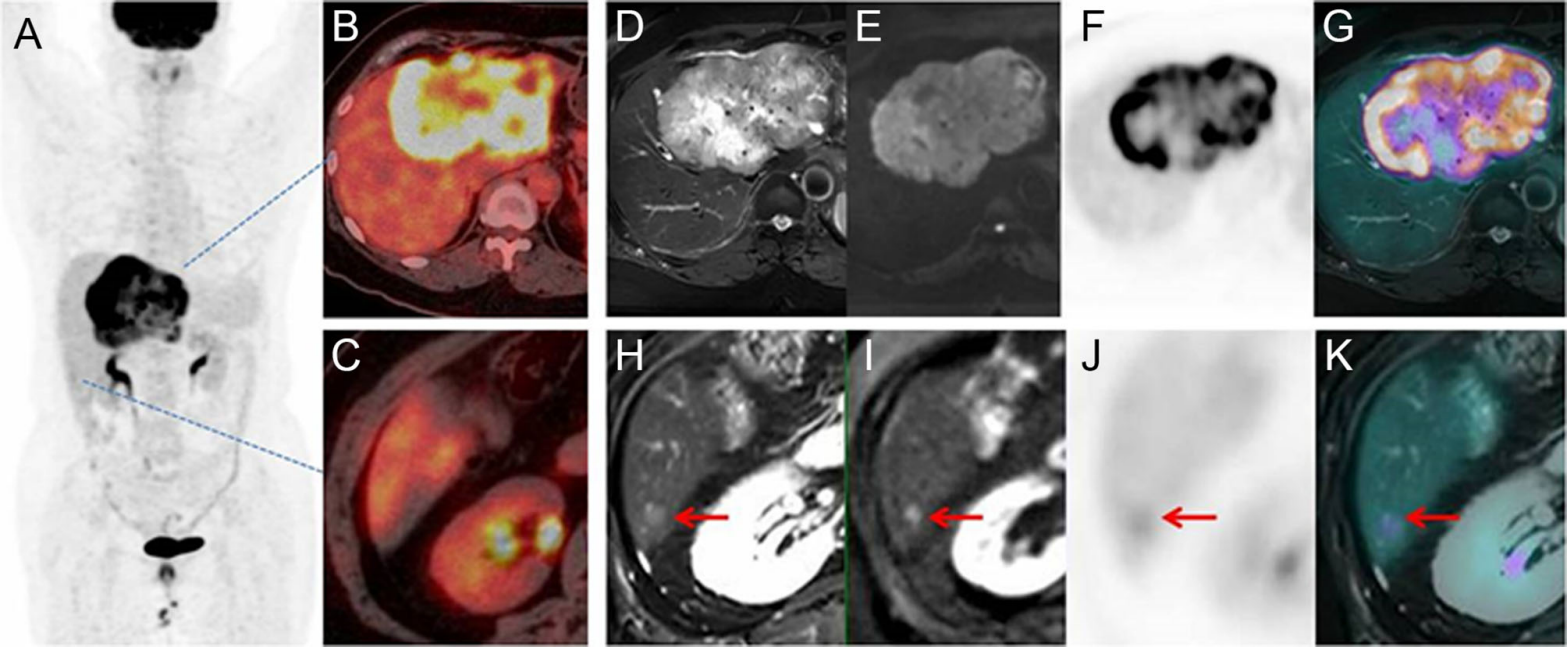
Images of a 63-year-old patient with liver mass. PET/CT MIP **(A)** and fusion image **(B, C)** showed a mass with high FDG uptake and no metastasis on the liver or distant organ. On PET/MR, the mass had hyperintensity on T2WI and DWI, with high FDG uptake on PET and fused image **(D–G)**. A 6-mm lesion was an additional finding by PET/MR **(H–K)**. Therefore, the stage was upgraded from M0 to M1.

As stated above, PET/MR detected distant metastasis in 13 patients who have been categorized as no distant metastasis by PET/CT, and a typical case is shown in **Figure 5**. Two of these patients were going through chemotherapy, and one patient was with mucinous adenocarcinoma of the colon. All these newly detected lesions were smaller than 10 mm (range, 4.0–10.0 mm; mean, 5.6 ± 1.4 mm).

However, PET/MR missed three metastasis lesions (diameter, 4, 7, and 8 mm, respectively), and none of these lesions had elevated [^18^F]FDG uptake or high DWI signal. All these lesions localized on the left lobe near the diaphragm (Segment 2) where the DWI signal was affected by the respiratory motion artifact.

## Discussion

In this study, we have conducted PET/CT and delayed PET/MR scans on patients with suspicious hepatic metastases and analyzed their diagnostic capability according to the reference standard.

The delayed PET/MR supplemented the diagnosis of PET/CT and improved the diagnostic accuracy of liver metastasis. This may exert an impact on therapeutic strategies. Take resectable colorectal cancer liver metastasis (CRLM), for example, the number of liver metastases determines the opportunity of surgical resection, as well as the need for preoperative chemotherapy. According to the National Comprehensive Cancer Network (NCCN) and European Society for Medical Oncology (ESMO) guidelines (22, 23), patients with a single metastasis ≤2 cm can be operated on directly, while other patients should receive neoadjuvant chemotherapy (24, 25). Three typical scenarios were identified as cases with altered therapeutic considerations: (a) PET/MR detected metastasis which was false negative in PET/CT; (b) PET/CT showed metastases in only one liver lobe, but PET/MR found metastases in both lobes; and (c) PET/CT gave false-positive findings, and PET/MR ruled them out. Out of the 70 patients enrolled, 29 fitted in these criteria. Each of these findings might possibly have an impact on the diagnosis and treatment (**Table 5**).

**Table 5:** Possible clinical impact of the delayed PET/MR in this study.

Type	Number of patients (%, *n* = 70)	Possible clinical impact
Detecting metastasis in PET/MR but not in PET/CT	13 (18.6%)	Changing the clinical staging
PET/CT showing metastasis in one lobe, while PET/MR showed metastases in 2 lobes	14 (20.0%)	Changing the surgical planning
PET/MR discovering the false-positive cases caused by PET/CT	2 (2.9%)	Changing the diagnosis
**Sum**	**29 (41.4%)**	

The diagnostic performance of PET/MR has also been evaluated in other clinical studies. Hybrid PET/MR with contrast enhancement showed higher accuracy for liver metastases (sensitivity, 92%–100%; specificity, 97%–100%) (26–28). This is consistent with the current findings (sensitivity, 97.4%; specificity, 100%) despite the different acquisition procedures, as this study did not involve contrast media (**Table 6**). In an earlier trial, Brendle et al. reported PET/MR (MR/DWI/PET) without contrast enhancement showed a relatively lower sensitivity (71%), specificity (80%), as well as diagnostic accuracy (74%) for liver metastases in colorectal cancer. This was mainly because the data contained a relatively high percentage of mucinous tumors, which are known to be challenging for both DWI and PET evaluation (29).

**Table 6:** Trials involving the comparison between PET/CT and PET/MR in liver metastasis.

	Trial 1^26^	Trial 2^27^	Trial 3^28^	This work
Purpose	PET/MR for metastases detection including liver	PET/MR for liver metastases detection		
Sample Size	15 colorectal cancer patients	41 patients with histologically confirmed solid tumors	32 patients with solid malignancies	70 patients with histologically confirmed solid tumors
Lesion number	37 lesions in the liver (15 benign, 22 malignant)	137 lesions in the liver (80 benign, 57 malignant)	113 lesions in the liver (68 benign, 45 malignant)	208 lesions in the liver (12 benign, 196 metastasis)
MR contrast	No	Gd-BOPTA	Gadovist	No
Sensitivity	0.71	0.98	0.92	0.98
Specificity	0.80	1.0	0.95	1.0
Accuracy	0.74	0.99	0.96	0.98
Discussion about lesion size	–	–	Mean diameter of metastases: 14 ± 8 mm	Mean lesion size detected by PET/MR is significantly smaller than that of PET/CT (*p* < 0.001).
Discussion about delayed PET	MR/DWI/PET showed higher accuracy than MR/DWI (0.74 *vs.* 0.52)	No statistically significant difference was shown between MR2 and PET/MR2 regarding the diagnostic confidence (*p* = 0.18)	SUVmax of lesions in PET/CT and PET/MR showed good correlation (*r* = 0.88; *p* < 0.001).	T/N was higher in PET/MR than PET/CT (5.0 ± 4.2 *vs.* 2.3 ± 1.1, *p* < 0.001). About 44.3% lesions had mild uptake (T/N ≤1.5) on the delayed PET.

Due to the limitations of the study protocol (single injection, double examination), PET/MR was performed after a longer injection interval (mean interval, 143 min) than PET/CT. Thus, the improved lesion detection ability is partly attributed to the delayed acquisition of PET. Since a longer interval leads to a higher lesion-to-background contrast (30, 31), more lesions showed [^18^F]FDG uptake in delayed PET, although part of them only showed mild contrast, which may have been missed without T2WI and DWI, or on a retrospective PET-MR fusion. Due to the difference in acquisition schemes, the PET acquisition time of PET/MR was much longer than that of PET/CT, which further improved the PET image quality, and enhanced the visibility of lesions with lower SUVs. As our results showed, lesions with smaller sizes were better identified with PET/MR, and the smallest lesion detected was 3 mm in size. Hence, a delayed and optimized PET/MR elevated diagnostic confidence. The combination of T2WI, T1WI, DWI, and the metabolic information from [^18^F]FDG has shown benefit for the detection of liver metastases. This illustrates the impact of multiparametric imaging in clinical diagnosis, which is in accordance with the findings of Beiderwellen et al. (26–28, 31–33).

In the current study, PET/CT showed a relatively low accuracy in lesion-based analysis, which is partly attributed to the bias of patient selection. Most patients with lesions that cannot be accurately diagnosed on PET/CT were selected. Meanwhile, the pathological type of the primary tumor (some tumor with low [^18^F]FDG uptake), and the process of chemotherapy (lesion activity suppression) influenced [^18^F]FDG uptake which needs comprehensive consideration for further evaluation.

The identification of the lesions could also be influenced by the difference in reconstruction parameters. Compared with PET/CT, PET/MR acquired more total counts, and the reconstruction parameters such as voxel spacing were not necessarily the same, and the PET image quality as characterized by noise and image resolution was higher in PET/MR compared with PET/CT. This discrepancy in image quality could partially account for the improved detection of smaller-sized lesions.

There are some limitations in our study. First, patients with different types of primary tumors have been enrolled, and their liver lesions exhibited different appearances. Second, patients with too many liver lesions have been excluded to facilitate lesion identification, thus the conclusions could not be simply extrapolated to these patients. Moreover, readers were not blinded to the history. Finally, histopathological confirmation of every detected lesion was not practical due to ethical and practical reasons.

It is noteworthy that the scope of this study is limited to the relative effect of delayed PET/MR scans of the liver. Further studies are required to elucidate the effect of PET/MR alone and to extend the conclusions to whole-body PET/MR scans.

## Conclusion

The diagnostic value of the delayed PET/MR in liver metastasis is validated and proved to be superior to that of PET/CT. The delayed PET/MR may promote the accurate identification of liver lesions and could improve the quality of clinical decision-making.

## Data Availability Statement

The raw data supporting the conclusions of this article will be made available by the authors, without undue reservation.

## Ethics Statement

The studies involving human participants were reviewed and approved by Medical Ethics Committee of Peking University Cancer Hospital. The patients/participants provided their written informed consent to participate in this study.

## Author Contributions

NZ and XM jointly designed the study and executed the protocols. NZ took the major responsibility in the study cohort, and XM in data analysis. BY assisted the data acquisition. JMY designed and conducted the MR-related parts. JYY was involved in the image evaluation. HZ and ZY jointly supervised the whole research process, conceptually designed the research ideas, and provided resources. All authors provided critical feedback and helped shape the research, analysis, and manuscript, and discussed the results. All authors contributed to the article and approved the submitted version.

## Funding

This work was financially supported by the National Science and Technology Major Project (No. 2020ZX09201023), National Natural Science Foundation (81871386), Beijing Municipal Administration of Hospitals - Yangfan Project (ZYLX201816), Beijing Millions of Talent Projects A level funding (No. 2019A38), and Science Foundation of Peking University Cancer Hospital (No. 2021-4).

## Conflict of Interest

The authors declare that the research was conducted in the absence of any commercial or financial relationships that could be construed as a potential conflict of interest.

## Publisher’s Note

All claims expressed in this article are solely those of the authors and do not necessarily represent those of their affiliated organizations, or those of the publisher, the editors and the reviewers. Any product that may be evaluated in this article, or claim that may be made by its manufacturer, is not guaranteed or endorsed by the publisher.
